# Adaptive Fuzzy Fixed-Time Trajectory Tracking Control for a Piezoelectric-Driven Microinjector

**DOI:** 10.3390/mi16121332

**Published:** 2025-11-26

**Authors:** Rungeng Zhang, Zehao Wu, Weijian Zhang, Qingsong Xu

**Affiliations:** Department of Electromechanical Engineering, Faculty of Science and Technology, University of Macau, Avenida da Universidade, Taipa, Macau, China; mc45206@um.edu.mo (R.Z.); zehaowu@um.edu.mo (Z.W.); yc27452@um.edu.mo (W.Z.)

**Keywords:** adaptive fuzzy logic, backstepping, fixed-time control, hysteresis nonlinearity, piezoelectric actuator

## Abstract

This paper proposes an adaptive fuzzy fixed-time control (AF-FxT-C) scheme for a piezoelectric-driven microinjector. The inherent hysteresis of the piezoelectric actuator is treated as an unknown nonlinearity. A fuzzy logic system is employed to approximate this hysteresis, along with other lumped disturbances, while an adaptive law is designed to improve approximation accuracy. To address the challenge of inconsistent initial states caused by frequent start-stop operations, a fixed-time control law is developed via a second-order backstepping approach. This guarantees that the upper bound of the system’s settling time is independent of the initial conditions, which is a claim rigorously substantiated by a theoretical stability analysis. The simulation and experimental results validate the effectiveness of the proposed method. The method also maintains robust tracking performance across reference signals of varying frequencies and amplitudes, demonstrating its potential for industrial microinjection applications.

## 1. Introduction

Piezoelectric actuators are widely applied in micro/nano manipulation due to their advantages of high precision, fast response, and compact size. In biomedical microinjection, numerous piezoelectric microinjectors capable of nanometer/micrometer precision have been developed [1,2,3]. However, these actuators exhibit inherent hysteresis nonlinearity, which complicates the system’s input-output relationship and degrades tracking performance [4]. A more critical challenge arises from the operational nature of microinjection, which involves frequent start-stop cycles. There is no guarantee that the injector will stop near the reference signal required for the next operation. This inconsistency in initial conditions poses a significant challenge for achieving rapid, stable, and precise control.

Existing control schemes for piezoelectric actuators are primarily divided into two categories. The first approach involves constructing an accurate model of the nonlinearity for feedforward compensation. The second treats the nonlinearity as a disturbance to be rejected via robust control strategies. The first approach has spurred extensive research into precise phenomenological models, such as the Preisach model [5], the Prandtl–Ishlinskii model [6], and the Bouc–Wen model [7]. For instance, Xiao et al. propose an improved inverse Preisach model that effectively compensates for rate-dependent hysteresis across a wide frequency range [8], while Habineza et al. extend the Bouc-Wen model to multivariable systems with strong cross-couplings and design efficient hysteresis compensators for multi-degree-of-freedom systems [9]. Meanwhile, there are already many models describing the rate-dependent characteristics of piezoelectric actuators. The authors of [10] propose using an elliptical family analytical model to describe the rate-dependent hysteresis characteristics of piezoelectric actuators, while reference [11] establishes a three-dimensional finite element model to simulate the rate-dependent domain change behavior of piezoelectric materials. These studies have greatly enriched the field of characteristic modeling for piezoelectric actuators. A critical limitation of this paradigm, however, is the inherent complexity of achieving precise modeling. The performance of model-based compensation is acutely dependent on model accuracy, which can be significantly degraded in practice by factors such as driving voltage frequency and operating temperature. Consequently, treating nonlinearities like hysteresis as unknown functions and employing robust, disturbance-rejecting control presents a more adaptive and practical alternative. Within this category, Fuzzy Logic Systems (FLSs) are widely adopted due to their universal approximation capabilities. These capabilities can be further enhanced through adaptive laws for dynamic online adjustment. For example, Chen et al. integrate an adaptive FLS into a backstepping control framework to approximate the actuator’s unknown efficiency factor and unmodeled nonlinearities (including hysteresis), enhancing the robustness and reliability of nanopositioning [12]. Similarly, Ghafarian et al. utilize an FLS within an Adaptive Fuzzy Sliding Mode Control (AFSMC) scheme to approximate system uncertainties and compensate for nonlinear effects (including hysteresis and disturbances), achieving high-precision robust tracking based on Lyapunov stability analysis [13]. For the hysteresis nonlinearity of piezoelectric micropositioning platforms, refs. [14,15] focus on the hysteresis problem of piezoelectric systems, using fuzzy/neural network methods to avoid constructing inverse hysteresis models.

Current research on piezoelectric actuator control predominantly focuses on minimizing steady-state tracking error, with comparatively less emphasis on the speed of the stabilization process itself. This is a critical oversight, as the inherent hysteresis nonlinearity in piezoelectric materials impedes the swift return of the system’s displacement to its original state after repeated actuations, thereby inducing static positioning errors. To accelerate system stabilization, the exploration of more effective control theories is essential. Finite-time control [16] has garnered significant interest for its ability to guarantee a bounded settling time, showing considerable promise for practical applications. A key limitation of this approach, however, is that its convergence time remains dependent on the system’s initial state. This dependency is problematic for piezoelectric microinjectors, which undergo frequent start-stop cycles where the initial position for the next operation is unpredictable and often far from the reference signal. This poses a major challenge for achieving rapid stabilization. To overcome this limitation, fixed-time control strategies [17] are particularly appealing, as they ensure an upper bound on the stabilization time that is independent of the initial conditions. While significant theoretical advances have been made in both finite-time [18,19,20,21,22,23,24] and fixed-time [25,26,27,28,29] control, these developments have largely remained within the theoretical domain or targeted at other applications. This paper presents an innovative contribution by applying fixed-time control to a piezoelectric microinjector for the first time, directly addressing the challenge of achieving rapid system stabilization regardless of the initial state.

In order to simultaneously solve the tracking error problem caused by hysteresis nonlinearity, this paper avoids cumbersome modeling compensation control methods and adopts an adaptive fuzzy logic system to approximate unknown nonlinearity, to enhance the robustness of the system. At the same time, in response to the problem of inconsistent initial conditions caused by frequent start stop operations, this paper adopts a backstepping method to design a fixed-time stable control law, which enables the system to have an independent upper bound on the convergence time independent of the initial state, especially suitable for piezoelectric microinjector systems. In summary, this article proposes a new adaptive fuzzy timing control (AF-FxT-C) strategy for piezoelectric microinjectors. This method synergistically integrates the unique advantages of fixed-time stability and adaptive fuzzy approximation:

- The fixed-time control framework guarantees that the system’s settling time is bounded by a constant independent of the initial conditions, thereby ensuring rapid transient performance across all operational scenarios.
- The adaptive fuzzy logic system obviates the need for precise explicit modeling of the complex hysteresis, enhancing robustness and environmental adaptability by online approximation of lumped unknown nonlinearities and disturbances.

A control law is meticulously constructed using a backstepping procedure, providing a rigorous theoretical guarantee of fixed-time stability. The feasibility and superior performance of the proposed strategy are conclusively demonstrated through both simulation and experimental validation on a piezoelectric microinjector system.

The remainder of this paper is organized as follows. Section 2 formulates the control problem. The proposed adaptive fuzzy fixed-time control strategy is developed in Section 3. The experimental setup is described in Section 4. The simulation and experimental results are presented and discussed in Section 5 and Section 6, respectively. Finally, concluding remarks are provided in Section 7.

## 2. Problem Formulation

### 2.1. Modeling of the Piezoelectric Microinjector

Referring to the model in [30], the state space model of the piezoelectric microinjector is derived as follows:

(1) $$\left\{\begin{array}{cc} \; & {\dot{x}}_{1}=x_{2}+f_{1}\\ \; & {\dot{x}}_{2}=-a_{0}x_{1}-a_{1}x_{2}+b_{0}u+f_{2}\\ \; & y=x_{1} \end{array}\right.$$

where $a_{0}=-\frac{K}{M}$, $a_{1}=-\frac{B}{M}$, $b_{0}=\frac{D}{M}$, *M* is the mass, *B* is damping coefficient, *K* is stiffness, and *x* represents the displacement response of the piezoactuated system. In addition, *D* is the piezoelectric coefficient and *u* denotes the input voltage. The terms $f_{1}$ and $f_{2}$ represent the nonlinear parts of the system model including model error and other disturbances, with $f_{2}$ specifically including the hysteresis nonlinearity of the system.

### 2.2. Lemmas and Assumption

**Lemma** **1** ([31]). *For a continuous unknown function f(Z) defined over a bounded closed set A, for ∀δ>0, there is the FLS $W^{T}G\left(Z\right)$ satisfying*

(2) $$\sup|f\left(Z\right)-W^{T}G\left(Z\right)|\leq \delta$$

*Here, uncertain functions with nonlinear terms are approximated by fuzzy logic system. We have*

(3) $$f\left(Z\right)=W^{T}G\left(Z\right)+\delta \left(Z\right)$$

*where |δ(Z)|≤ε, with δ(Z) being an estimation error and ε>0 being a parameter. $W={\left[\omega_{1},\omega_{2}\right]}^{T}$ denotes the desired weight vector. $G\left(Z\right)=\frac{{\left[h_{1}\left(Z\right),h_{2}\left(Z\right),\ldots ,h_{m}\left(Z\right)\right]}^{T}}{\sum_{i=1}^{m}h_{i}\left(Z\right)}$ represents the basis function vector, with m>1 being the rule number, and $h_{i}\left(Z\right)=\exp\left(-\frac{{\left(Z_{i}-v_{i}\right)}^{T}\left(Z_{i}-v_{i}\right)}{e_{i}^{T}e_{i}}\right)$ describing the Gaussian function. In addition, $v_{i}=\left[v_{i1},v_{i2},\ldots ,v_{in}\right]$ denotes the center vector, and $e_{i}=\left[e_{i1},e_{i2},\ldots ,e_{in}\right]$ represents the width of the Gaussian function.*

**Lemma** **2** ([32]). *For a given constant O>0 and x∈R, we have*

(4) $$0\leq \left|x\right|-x\tanh\left(\frac{x}{O}\right)\leq \rho O$$

*where $\rho =\sup_{t>0}\left(\frac{1}{1+e^{t}}\right)=0.2785$.*

**Lemma** **3** ([33]). *Choosing $\zeta_{1}>0$, $\zeta_{2}>0$, $\zeta_{3}>0$, $\psi_{1}\geq 0$, $\psi_{2}\geq 0$, and $\psi_{3}\geq 0$, we can obtain*

(5) $$\begin{array}{cc} \psi_{1}^{\zeta_{1}}\psi_{2}^{\zeta_{2}} & \leq \zeta_{3}\psi_{1}^{\zeta_{1}+\zeta_{2}}+\frac{\zeta_{1}}{\zeta_{1}+\zeta_{2}}\\ \; & \;\times {\left(\frac{\zeta_{1}}{\zeta_{3}\left(\zeta_{1}+\zeta_{2}\right)}\right)}^{\frac{\zeta_{1}}{\zeta_{2}}}\psi_{2}^{\zeta_{1}+\zeta_{2}}\psi_{3}^{\frac{\zeta_{1}+\zeta_{2}}{\zeta_{2}}} \end{array}$$

**Lemma** **4** ([34]). *For $\varpi_{1},\varpi_{2}\in R$, we have*

(6) $$\varpi_{1}\varpi_{2}\leq \frac{\epsilon^{m}}{m}|\varpi_{1}{|}^{m}+\frac{1}{n\epsilon^{n}}{\left|\varpi_{2}\right|}^{n}$$

*with ϵ>0, m>1, n>1, and (m−1)(n−1)=1.*

**Lemma** **5** ([35]). *If $x_{1},x_{2},\ldots ,x_{n}\geq 0$ and 0<α<1, then*

(7) $$\sum_{i=1}^{n}x_{i}^{\alpha }\geq {\left(\sum_{i=1}^{n}x_{i}\right)}^{\alpha }.$$

**Lemma** **6** ([36]). *For $\tau_{i}>0$, i=1,…,m, we get*

(8) $${\left(\sum_{i=1}^{m}\tau_{i}\right)}^{2}\leq m\sum_{i=1}^{m}\tau_{i}^{2}$$

**Lemma** **7** ([37]). *Assuming the existence of a positive definite function V(ξ) and parameters a,b>0, 0<λ<1, and 1<μ<+∞, such that $\xi (t,\xi_{0})$ satisfies:*

(9) $$\dot{V}\left(\xi \right)\leq -aV^{\lambda }\left(\xi \right)-bV^{\mu }\left(\xi \right)+\varrho ,\;\varrho >0$$

*Then, the nonlinear system is a semi-global practical fixed-time stable system, and its settling time satisfies:*

(10) $$T\left(\xi \right)\leq T_{\max}=\frac{2}{a(1-\lambda )}+\frac{2}{b(\mu -1)}$$

**Assumption** **1.** *All signals within a closed-loop system are bounded. The reference signal $y_{r}$ and its first-order derivative are bounded and continuous.*

In practical applications of piezoelectric actuators (PEAs), it is essential to keep the reference signal within a bounded range to avoid potential damage to the system. This boundedness of the reference signal and its derivative is also a common assumption in backstepping-based nonlinear control methods. For the purpose of this research, considering ease of implementation and the need for a representative trajectory, a sinusoidal signal is chosen as the reference trajectory in simulation and experimental studies.

## 3. Adaptive Fuzzy Fixed-Time Controller Design

**Figure 1:**
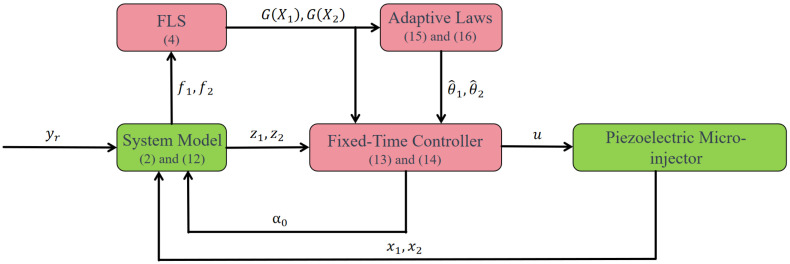
Control block diagram for the piezoelectric microinjector with adaptive fuzzy fixed-time control method.

This section details the development of an adaptive fuzzy fixed-time control (AF-FxT-C) strategy. The control objective is to ensure the system’s output displacement accurately tracks a given reference signal. The corresponding control diagram, illustrating the architecture and parameter flow, is presented in Figure 1.

Define the following coordinate transformations:

(11) $$\left\{\begin{array}{c} z_{1}=x_{1}-y_{r},\\ z_{2}=x_{2}-\alpha_{0}, \end{array}\right.$$

where $y_{r}$ is the reference signal, $z_{i}\;\left(i=1,2\right)$ is the tracking error, and $\alpha_{0}$ is the virtual control law.

**Theorem** **1.** *Consider the second-order system plant represented by (1). Under the control law (12) and (13) and the adaptive laws (14) and (15), the proposed adaptive fuzzy fixed-time control scheme guarantees that the system settling time is uniformly bounded by $T_{\max}$ in (36), independent of the initial conditions.*

The virtual control law is given as follows:

(12) $$\begin{array}{cc} \alpha_{0}= & -K_{11}\tanh\left(\frac{z_{1}}{o_{1}}\right)-K_{12}z_{1}^{3}\\ \; & -\frac{1}{2\iota_{1}^{2}}z_{1}{\hat{\theta }}_{1}G^{T}\left(X_{1}\right)G\left(X_{1}\right)-\frac{1}{2}z_{1} \end{array}$$

The final control law is represented as follows:

(13) $$\begin{array}{cc} u & =\frac{1}{b_{0}}\left(-K_{21}\tanh\left(\frac{z_{2}}{O_{2}}\right)-K_{22}z_{2}^{3}-z_{1}+a_{0}x_{1}\right.\\ \; & \;\left.+a_{1}x_{2}-\frac{1}{2\iota_{2}^{2}}z_{2}{\hat{\theta }}_{2}G^{T}\left(X_{2}\right)G\left(X_{2}\right)-\frac{1}{2}z_{2}\right) \end{array}$$

where $K_{11},K_{12},K_{21},K_{22},O_{1},O_{2},\iota_{1},\mathrm{and}\;\iota_{2}$ are positive setting parameters. $X_{1}=x_{1}$, $X_{2}={\left[x_{1},x_{2}\right]}^{T}$. ${\hat{\theta }}_{i}$ is an estimate of the adaptive law, with ${\tilde{\theta }}_{i}=\theta_{i}-{\hat{\theta }}_{i}$ and $\theta_{i}=W_{i}^{T}W_{i}$ (i=1,2).

The update rule for the estimated values of the adaptive law is denoted below.

(14) $${\dot{\hat{\theta }}}_{1}=\frac{1}{2\iota_{1}^{2}}z_{1}^{2}G^{T}\left(X_{1}\right)G\left(X_{1}\right)-p_{1}{\hat{\theta }}_{1}-q_{1}{\hat{\theta }}_{1}^{3}$$

(15) $${\dot{\hat{\theta }}}_{2}=\frac{1}{2\iota_{2}^{2}}z_{2}^{2}G^{T}\left(X_{2}\right)G\left(X_{2}\right)-p_{2}{\hat{\theta }}_{2}-q_{2}{\hat{\theta }}_{2}^{3}$$

where $p_{1},q_{1},p_{2},\mathrm{and}\;q_{2}$ are positive setting parameters.

**Proof.** **Step 1.** Selecting the following Lyapunov function:

(16) $$V_{1}=\frac{1}{2}z_{1}^{2}+\frac{1}{2}{\tilde{\theta }}_{1}^{2}$$

The time differentiation of (16) yields

(17) $$\begin{array}{cc} {\dot{V}}_{1} & =z_{1}\left(x_{2}+f_{1}-{\dot{y}}_{r}\right)-{\tilde{\theta }}_{1}{\dot{\hat{\theta }}}_{1}\\ \; & =z_{1}\left(z_{2}+\alpha_{0}+f_{1}-{\dot{y}}_{r}\right)-{\tilde{\theta }}_{1}{\dot{\hat{\theta }}}_{1} \end{array}$$

Define ${\bar{f}}_{1}=f_{1}-{\dot{y}}_{r}$, $\forall \varepsilon_{1}>0$, according to Lemma 1, ${\bar{f}}_{1}=W_{1}^{T}G\left(Z_{1}\right)+\delta \left(Z_{1}\right)$, where $|\delta \left(Z_{1}\right)|<\varepsilon_{1}^{*}$ with $Z_{2}={\left[x_{1},y_{r},{\dot{y}}_{r}\right]}^{T}$.

According to Young’s inequality, the following conclusion can be drawn.

(18) $$z_{1}\bar{f_{1}}\leq \frac{1}{2\iota_{1}^{2}}z_{1}^{2}\theta_{1}G^{T}\left(X_{1}\right)G\left(X_{1}\right)+\frac{1}{2}\iota_{1}^{2}+\frac{1}{2}z_{1}^{2}+\frac{1}{2}\varepsilon_{1}^{*2}$$

where $X_{1}=x_{1}\in R$.

By summarizing (17) and (18), scaling the derivative of the Lyapunov function, it is obtained that

(19) $$\begin{array}{cc} {\dot{V}}_{1}\leq & z_{1}\left(z_{2}+\alpha_{0}+\frac{1}{2}z_{1}\right.\\ \; & \left.+\frac{1}{2\iota_{1}^{2}}z_{1}{\hat{\theta }}_{1}G^{T}\left(X_{1}\right)G\left(X_{1}\right))\right.\\ \; & \left.+{\tilde{\theta }}_{1}\left(\frac{1}{2\iota_{1}^{2}}z_{1}^{2}G^{T}\left(X_{1}\right)G\left(X_{1}\right)\right)-{\dot{\hat{\theta }}}_{1}\right)\\ \; & +\frac{1}{2}\iota_{1}^{2}+\frac{1}{2}\varepsilon_{1}^{*2} \end{array}$$

where the virtual control law $\alpha_{0}$ is defined by (13), and the update law ${\dot{\hat{\theta }}}_{2}$ is given by (15).

Combining (12), (14) and (19), and Lemma 2, it is obtained that

(20) $$\begin{array}{cc} {\dot{V}}_{1}\leq & -K_{11}{\left(z_{1}^{2}\right)}^{\frac{1}{2}}-K_{12}{\left(z_{1}^{2}\right)}^{2}+z_{1}z_{2}\\ & +p_{1}{\tilde{\theta }}_{1}{\hat{\theta }}_{1}+q_{1}{\tilde{\theta }}_{1}{\hat{\theta }}_{1}^{3}+O_{1}\rho +\frac{1}{2}\iota_{1}^{2}+\frac{1}{2}\varepsilon_{1}^{*2} \end{array}$$

In view of Young’s inequality, it is true that

(21) $$p_{1}{\tilde{\theta }}_{1}{\hat{\theta }}_{1}\leq -\frac{1}{2}p_{1}{\tilde{\theta }}_{1}^{2}+\frac{1}{2}p_{1}\theta_{1}^{2}$$

According to Lemma 3, we can obtain

(22) $${\left(\frac{p_{1}{\tilde{\theta }}_{1}^{2}}{2}\right)}^{\frac{1}{2}}\leq \left(1-\frac{1}{2}\right){\left(\frac{1}{2}\right)}^{\frac{1}{2}/\left(1-\frac{1}{2}\right)}+\frac{p_{1}{\tilde{\theta }}_{1}^{2}}{2}$$

Because of ${\tilde{\theta }}_{1}{\tilde{\theta }}_{1}^{3}={\tilde{\theta }}_{1}{\left(\theta_{1}-{\tilde{\theta }}_{1}\right)}^{3}={\tilde{\theta }}_{1}\left(\theta_{1}^{3}-3\theta_{1}^{2}{\tilde{\theta }}_{1}+3\theta_{1}{\tilde{\theta }}_{1}^{2}-{\tilde{\theta }}_{1}^{3}\right)$, through Lemma 4, we can derive

(23) $$\begin{array}{cc} 3q_{1}{\tilde{\theta }}_{1}^{3}\theta_{1} & \leq \frac{9q_{1}\epsilon^{\frac{4}{3}}{\tilde{\theta }}_{1}^{4}}{4}+\frac{3q_{1}\theta_{1}^{4}}{4\epsilon^{4}}\\ q_{1}{\tilde{\theta }}_{1}\theta_{1}^{3} & \leq 3q_{1}{\tilde{\theta }}_{1}^{2}\theta_{1}^{2}+\frac{q_{1}\theta_{1}^{4}}{12} \end{array}$$

Based on (21)–(23), the Lyapunov function can be scaled as follows.

(24) $$\begin{array}{cc} {\dot{V}}_{1}\leq & -K_{11}{\left(z_{1}^{2}\right)}^{\frac{1}{2}}-K_{12}{\left(z_{1}^{2}\right)}^{2}-{\left(\frac{p_{1}{\tilde{\theta }}_{1}^{2}}{2}\right)}^{\frac{1}{2}}\\ \; & -\left(4q_{1}-9q_{1}\epsilon^{\frac{4}{3}}\right){\left(\frac{{\tilde{\theta }}_{1}^{2}}{2}\right)}^{2}+z_{1}z_{2}+\Delta_{1} \end{array}$$

where $\Delta_{1}=O_{1}\rho +\frac{1}{2}\iota_{1}^{2}+\frac{1}{2}\varepsilon_{1}^{*2}+\frac{p_{1}\theta_{1}^{2}}{2}+\frac{3q_{1}\theta_{1}^{4}}{4\epsilon^{4}}+\frac{q_{1}\theta_{1}^{4}}{12}$.

**Step 2.** The Lyapunov function is chosen as follows:

(25) $$V_{2}=V_{1}+\frac{1}{2}z_{2}^{2}+\frac{1}{2}{\tilde{\theta }}_{2}^{2}$$

Then, it is obtained that

(26) $$\begin{array}{cc} {\dot{V}}_{2}\leq & -K_{11}{\left(z_{1}^{2}\right)}^{\frac{1}{2}}-K_{12}{\left(z_{1}^{2}\right)}^{2}-{\left(\frac{p_{1}{\tilde{\theta }}_{1}^{2}}{2}\right)}^{\frac{1}{2}}\\ \; & -\left(4q_{1}-9q_{1}\epsilon^{\frac{4}{3}}\right){\left(\frac{{\tilde{\theta }}_{1}^{2}}{2}\right)}^{2}+z_{1}z_{2}+\Delta_{1}\\ \; & +z_{2}\left(-a_{0}x_{1}-a_{1}x_{2}+b_{0}u+f_{2}-{\dot{\alpha }}_{0}\right)-{\tilde{\theta }}_{2}{\dot{\hat{\theta }}}_{2} \end{array}$$

Defining ${\bar{f}}_{2}=f_{2}-{\dot{\alpha }}_{0}$, $\forall \varepsilon_{2}>0$, ${\bar{f}}_{2}=W_{2}^{T}G\left(Z_{2}\right)+\delta \left(Z_{2}\right)$, where $|\delta \left(Z_{2}\right)|<\varepsilon_{2}^{*}$ and $Z_{2}={\left[x_{1},x_{2},{\hat{\theta }}_{1},y_{r},{\dot{y}}_{r}\right]}^{T}$.

Similarly to (18), one has

(27) $$z_{2}{\bar{f}}_{2}\leq \frac{1}{2\iota_{2}^{2}}z_{2}^{2}\theta_{2}G^{T}\left(X_{2}\right)G\left(X_{2}\right)+\frac{1}{2}\iota_{2}^{2}+\frac{1}{2}z_{2}^{2}+\frac{1}{2}\varepsilon_{2}^{*2}$$

where $X_{2}=x_{2}\in R$.

In view of Equations (26) and (27), we can obtain

(28) $$\begin{array}{cc} {\dot{V}}_{2}\leq & -K_{11}{\left(z_{1}^{2}\right)}^{\frac{1}{2}}-K_{12}{\left(z_{1}^{2}\right)}^{2}-{\left(\frac{p_{1}{\tilde{\theta }}_{1}^{2}}{2}\right)}^{\frac{1}{2}}\\ \; & -\left(4q_{1}-9q_{1}\epsilon^{\frac{4}{3}}\right){\left(\frac{{\tilde{\theta }}_{1}^{2}}{2}\right)}^{2}+\Delta_{1}\\ \; & +z_{2}(-a_{0}x_{1}-a_{1}x_{2}+b_{0}u+z_{1}+\frac{1}{2}z_{2}\\ \; & +\frac{1}{2l_{2}^{2}}z_{2}{\hat{\theta }}_{2}G^{T}\left(X_{2}\right)G\left(X_{2}\right))\\ \; & -{\tilde{\theta }}_{2}\left(\frac{1}{2l_{2}^{2}}z_{2}^{2}G^{T}\left(X_{2}\right)G\left(X_{2}\right)-{\dot{\hat{\theta }}}_{2}\right)\\ \; & +\frac{1}{2}l_{2}^{2}+\frac{1}{2}\varepsilon_{2}^{*2} \end{array}$$

Then, given the input *u* in (13) and the update law ${\dot{\hat{\theta }}}_{2}$ in (15), combining (13), (15), and (28), the following scaling transformations can be derived.

(29) $$\begin{array}{cc} {\dot{V}}_{2}\leq & -K_{11}{\left(z_{1}^{2}\right)}^{\frac{1}{2}}-K_{12}{\left(z_{1}^{2}\right)}^{2}-{\left(\frac{p_{1}{\tilde{\theta }}_{1}^{2}}{2}\right)}^{\frac{1}{2}}\\ \; & -\left(4q_{1}-9q_{1}\epsilon^{\frac{4}{3}}\right){\left(\frac{{\tilde{\theta }}_{1}^{2}}{2}\right)}^{2}+\Delta_{1}\\ \; & -K_{21}{\left(z_{2}^{2}\right)}^{\frac{1}{2}}-K_{22}{\left(z_{2}^{2}\right)}^{2}+p_{2}{\tilde{\theta }}_{2}{\hat{\theta }}_{2}\\ \; & +q_{2}{\tilde{\theta }}_{2}{\hat{\theta }}_{2}^{3}+\frac{1}{2}\iota_{2}^{2}+\frac{1}{2}\varepsilon_{2}^{*2} \end{array}$$

According to the Equations (22)–(24), we have

(30) $$\begin{array}{cc} {\dot{V}}_{2} & \leq -\sum_{j=1}^{2}K_{j1}{\left(z_{j}^{2}\right)}^{\frac{1}{2}}-\sum_{j=1}^{2}K_{j2}{\left(z_{j}^{2}\right)}^{2}-\sum_{j=1}^{2}{\left(\frac{p_{j}{\tilde{\theta }}_{j}^{2}}{2}\right)}^{\frac{1}{2}}\\ \; & \;-\sum_{j=1}^{2}\left(4q_{j}-9q_{j}\epsilon^{\frac{4}{3}}\right){\left(\frac{{\tilde{\theta }}_{j}^{2}}{2}\right)}^{2}+\Delta_{2} \end{array}$$

where $\Delta_{2}=\Delta_{1}+O_{2}\rho +\frac{1}{2}\iota_{2}^{2}+\frac{1}{2}\varepsilon_{2}^{*2}+\frac{p_{2}\theta_{2}^{2}}{2}+\left(1-\frac{1}{2}\right){\left(\frac{1}{2}\right)}^{\frac{1}{2}/\left(1-\frac{1}{2}\right)}+\frac{3q_{2}\theta_{2}^{4}}{4\epsilon^{4}}+\frac{q_{2}\theta_{2}^{4}}{12}$.

In view of (23), we have

(31) $$\begin{array}{cc} {\dot{V}}_{2}\leq & -\sum_{j=1}^{2}2^{\frac{1}{2}}K_{j1}{\left(\frac{z_{j}^{2}}{2}\right)}^{\frac{1}{2}}-\sum_{j=1}^{2}2^{2}K_{j2}{\left(\frac{z_{j}^{2}}{2}\right)}^{2}\\ \; & -\sum_{j=1}^{2}p_{j}^{\frac{1}{2}}{\left(\frac{{\hat{\theta }}_{j}^{2}}{2}\right)}^{\frac{1}{2}}\\ \; & -\sum_{j=1}^{2}\left(4q_{j}-9q_{j}\epsilon^{\frac{4}{3}}\right){\left(\frac{{\hat{\theta }}_{j}^{2}}{2}\right)}^{2}+\Delta_{2} \end{array}$$

Defining $\Gamma_{1}=\min\left(2^{\frac{1}{2}}K_{11},2^{\frac{1}{2}}K_{21},p_{1}^{\frac{1}{2}},p_{2}^{\frac{1}{2}}\right)$ and ${\overset{˘}{\Gamma }}_{2}=\min\left(2^{2}K_{12},2^{2}K_{22},4q_{1}-9q_{1}\epsilon^{\frac{4}{3}},4q_{2}-9q_{2}\epsilon^{\frac{4}{3}}\right)$, with $\Gamma_{1}>0$ and ${\overset{˘}{\Gamma }}_{2}>0$, we can obtain

(32) $$\begin{array}{cc} {\dot{V}}_{2}\leq & -\Gamma_{1}\left(\sum_{j=1}^{2}{\left(\frac{z_{j}^{2}}{2}\right)}^{\frac{1}{2}}+\sum_{j=1}^{2}{\left(\frac{{\tilde{\theta }}_{j}^{2}}{2}\right)}^{\frac{1}{2}}\right)\\ \; & -{\overset{˘}{\Gamma }}_{2}\left(\sum_{j=1}^{2}{\left(\frac{z_{j}^{2}}{2}\right)}^{2}+\sum_{j=1}^{2}{\left(\frac{{\tilde{\theta }}_{j}^{2}}{2}\right)}^{2}\right)+\Delta_{2} \end{array}$$

Considering Lemma 5 and Lemma 6, it is true that

(33) $$V_{2}^{\frac{1}{2}}\leq \sum_{j=1}^{2}{\left(\frac{z_{j}^{2}}{2}\right)}^{\frac{1}{2}}+\sum_{j=1}^{2}{\left(\frac{{\tilde{\theta }}_{j}^{2}}{2}\right)}^{\frac{1}{2}}$$

(34) $$V_{2}^{2}\leq 2\times 2\left(\sum_{j=1}^{2}{\left(\frac{z_{j}^{2}}{2}\right)}^{2}+\sum_{j=1}^{2}{\left(\frac{{\hat{\theta }}_{j}^{2}}{2}\right)}^{2}\right)$$

Combining Equations (25), (26), and (27) yields

(35) $${\dot{V}}_{2}\leq -\Gamma_{1}V_{2}^{\frac{1}{2}}-\Gamma_{2}V_{2}^{2}+\Delta_{2}$$

where $\Gamma_{2}=\frac{{\overset{˘}{\Gamma }}_{2}}{4}$.

Using Lemma 7, we can obtain the upper bound on the settling time:

(36) $$T_{\max}=\frac{4}{\Gamma_{1}}+\frac{2}{\Gamma_{2}}$$

Ultimately, it can be proven that the system is fixed-time stable, and the upper bound of settlement time is (36). □

**Remark** **1.** *Unlike finite-time control, the stability condition for fixed-time control does not contain terms dependent on the system’s initial states. The finite-time control law used for experimental comparison was derived by modifying the proposed fixed-time control law; specifically, the 3rd-power terms in both the control and adaptive laws were replaced with 0.9th-power terms. Consequently, the proposed controller guarantees a consistent upper bound on the settling time (36), regardless of initial conditions. This property is particularly advantageous for piezoelectric microinjectors, which require rapid stabilization during frequent start-stop reciprocating motions characterized by hysteresis nonlinearity. In such applications, it is difficult to ensure that the initial position is near the reference signal upon restarting after each stop. For further comparison, a fixed-time sliding mode control (SMC) law from [38] was also implemented. However, the chattering phenomenon inherent to classical SMC presents a significant drawback, making it difficult to deploy on practical hardware.*

**Remark** **2.** *Building upon the research in [21], the integration of a hyperbolic tangent function into the control law design serves a dual purpose: it fundamentally eliminates the risk of singularity during the differentiation of virtual control laws and simultaneously suppresses chattering in the control voltage. This mitigation of high-frequency oscillations is critical for preventing potential damage to the piezoelectric ceramics.*

**Remark** **3.** *An adaptive fuzzy logic system (FLS) is employed to approximate the unknown nonlinearities, thereby circumventing the need for cumbersome and precise hysteresis modeling. This approach prevents performance degradation due to model inaccuracies arising from external variations, significantly enhancing the system’s robustness and adaptability. Gaussian functions are selected as membership functions, and their centers and standard deviations must be carefully chosen by the designer to ensure they adequately cover the entire operational range of the approximated variables.*

**Remark** **4.** *Theoretical analysis, supported by simulations and experiments, demonstrates that increasing the control gains ($K_{11}$, $K_{12}$, $K_{21}$, $K_{22}$, $p_{1}$, and $p_{2}$) reduces the upper bound of the settling time. However, excessively high gains may induce undesirable system oscillations. Furthermore, the settling time is also influenced by the parameters $q_{1}$, $q_{2}$, and the inherent approximation errors of the fuzzy logic system (FLS). Consequently, these parameters must be meticulously tuned to achieve an optimal balance between rapid convergence and system stability.*

**Remark** **5.** *Fixed-time control guarantees a settling-time bound independent of initial conditions, which may lead to large control inputs during the initial transient. This poses potential challenges for practical implementation in piezoelectric actuators. However, in the present application, the initial state deviation is constrained within the actuator’s physical stroke limit. As a result, the control input remains within a reasonable range and does not exceed the hardware’s operational limits. Moreover, although hysteresis in piezoelectric actuators is inherently an input-dependent nonlinearity, it is treated here as part of a lumped disturbance term. This modeling approach, adopted from [39], simplifies the controller design and stability analysis while maintaining effectiveness in practice.*

## 4. Prototyping and Experimental Setup

### 4.1. Development of the Microinjector Prototype

**Figure 2:**
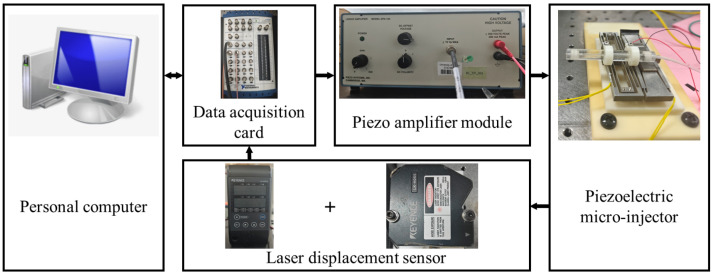
Experimental setup of a piezoelectric microinjector system.

A photograph of the experimental prototype is presented in Figure 2. The core actuating element of the piezo-driven microinjector is a Lead Zirconate Titanate (PZT) ceramic block. It is a P-888.91 piezoelectric transducer from PI company (Karlsruhe, Germany). The recommended preload and maximum preload for PZT are 15 MPa and 30 MPa, respectively. Integrated with PZT is a mechanical displacement amplification structure. This assembly forms a second-order system with a centrally fixed syringe needle, capable of an output displacement of up to 500 µm.

The output displacement is measured by a KEYENCE LK-HD500 laser displacement sensor (KEYENCE Corporation, Osaka, Japan). The resulting signal is acquired differentially by a 16-bit National Instruments (NI) USB-6259 data acquisition (DAQ) card (National Instruments Corporation, Austin, TX, USA). Control laws are computed in MATLAB R2023a (The MathWorks, Inc., Natick, MA, USA) on a host PC, which generates the control signals. These signals are output through the same DAQ card to a Piezo Systems EPA-104 voltage amplifier (Piezo Systems, Inc., Woburn, MA, USA). The amplifier provides a gain of 20, boosting the control signal before driving the microinjector, thereby closing the control loop.

### 4.2. System Identification

**Figure 3:**
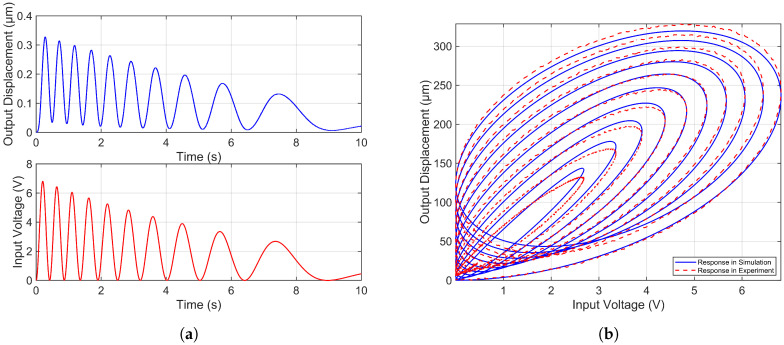
(**a**) Input and response of the piezoelectric microinjector in system identification. (**b**) Experimental and simulation responses of the piezoelectric microinjector system under test signal.

A cosine signal (37), with simultaneously decaying amplitude and phase, is used to excite the system. The resulting displacement output is recorded using a laser displacement sensor. The system input and corresponding response are shown in Figure 3a. This input-output data is then used to perform a linear model identification in the time domain via the MATLAB System Identification Toolbox. The identified model is represented as a transfer function with two poles and no zeros. It is important to note that the dynamics of the piezoelectric signal amplifier are included as part of the system during this identification process.

(37) $$u_{\mathrm{test}}=3.5e^{-0.13t}\left[\cos\left(5\pi t\times e^{-0.09t}-3.15\right)+1.0\right]$$

The transfer function model is identified as follows.

(38) $$G\left(s\right)=\frac{0.02676}{s^{2}+40.45s+497.6}\;\left(m/V\right)$$

where *s* is the Laplace operator. Equating the coefficients of (1) and (38), the parameters of the dynamic model are calculated as: $a_{1}=40.45$, $a_{0}=497.6$, and $b_{0}=0.02676$, respectively.

For control simulation purposes, the Bouc-Wen model was selected to characterize the hysteresis of the piezoelectric microinjector. This model is favored for its simple mathematical form and its ability to explicitly represent hysteresis nonlinearity. The model is described by the following equations:

(39) $$\dot{H}=\alpha D\dot{u}-\beta |\dot{u}|H-\gamma \dot{u}\left|H\right|$$

where *H* indicates the hysteretic loop in terms of displacement whose magnitude and shape are determined by parameters α,β, and γ.

Using the preidentified linear system and input-output data, the hysteresis model parameters D,α,β, and γ were optimized via a particle swarm algorithm targeting error minimization. Finally, the hysteresis parameters of the system under the Bouc-Wen model are derived as: $D=1.0\times 10^{-5}\;m/V$, α=0.12, β=3.1, and γ=−2.8.

Figure 3b compares the experimental response of the piezoelectric microinjector to the input signal (37) with the simulated output of the identified system model. The close agreement between the model output and the experimental data validates the success of the system identification process.

## 5. Simulation Study

This section details the simulation of sinusoidal trajectory tracking control using the proposed adaptive fuzzy fixed-time control (AF-FxT-C) strategy. Based on the model obtained from system identification, the nonlinear components of the system are constructed. The simulations are performed using a fourth-order Runge-Kutta method with a fixed step size of 0.001.

We evaluate the tracking performance graphically and simultaneously monitor the evolution of the adaptive parameters and the control voltage. The implementation of system (1) in the simulation environment is detailed below:

(40) $$\begin{array}{ccc} f_{1} & = & 0.1\sin\left(10x_{1}\right)+0.1\sin\left(10x_{2}\right) \end{array}$$

(41) $$\begin{array}{ccc} f_{2} & = & H+0.1\sin\left(10x_{1}\right)+0.1\sin\left(10x_{2}\right) \end{array}$$

The sine interference in $f_{1}$ and $f_{2}$ is an idealized nonlinear test function used to verify the robustness of the controller. Its frequency was deliberately set equal to that of the reference signal so as to mimic the harmonic disturbances most likely to be excited during tracking experiments. The control parameters, reference signal, initial values of the adaptive laws, and initial states used in the simulation are summarized in Table 1.

**Figure 4:**
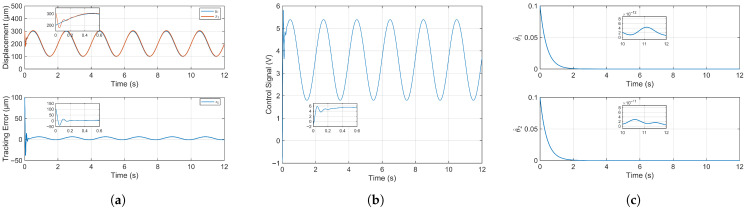
Simulation results. (**a**) Trajectory tracking control results and tracking error of simulation study. (**b**) Control signal in simulation study. (**c**) Estimation value of adaptive law in simulation study.

Figure 4a presents the trajectory tracking performance of the system under the proposed adaptive fuzzy fixed-time control. Despite an initial position offset of 100 µm, the piezoelectric microinjector achieves stable tracking. The root mean square error (RMSE) is 10.5191 µm in the first cycle and reduces to 3.7509 µm thereafter, demonstrating rapid convergence. An enlarged view of the initial transient confirms the system’s ability to quickly reach and track the reference signal. The stable RMSE constitutes merely 1.88% of the system’s total motion range, indicating highly accurate and successful tracking control.

The temporal evolution of the control signal is depicted in Figure 4b. A characteristic rapid and substantial variation is observed during the initial transient period, which is a hallmark of the fixed-time control strategy. This aggressive initial actuation is intentionally designed to force the system to converge within a predetermined time bound, independent of the initial conditions. Following this transient phase, the control signal stabilizes into smooth, sinusoidal oscillations. This stable, periodic pattern demonstrates the controller’s ability to generate the precise control voltage necessary for the system to accurately track the sinusoidal reference signal while ensuring closed-loop stability.

The temporal evolution of the adaptive law’s estimated value is illustrated in Figure 4c. Initialized at 0.1, the parameter estimates converge gradually to the order of $10^{-12}$ and $10^{-11}$, respectively. The adaptive law estimation value only guarantees boundedness and ultimately hovers around a constant value, which is in line with theory. Crucially, the adaptive laws remain bounded throughout the entire process, demonstrating the stability of the adaptation mechanism.

## 6. Experimental Results and Discussion

This section details the experimental validation of the adaptive fuzzy fixed-time control (AF-FxT-C) strategy for trajectory tracking. Utilizing the experimental setup shown in Figure 2, a closed-loop control platform was constructed. All experiments were conducted at a sampling frequency of 500 Hz, corresponding to a control step size of 2 ms. The control parameters used are provided in Table 2.

### 6.1. Trajectory Tracking with AF-FxT-C

The system was tasked with tracking a sinusoidal reference signal characterized by a 100 µm amplitude, a 200 µm DC bias, and a frequency of 0.5 Hz, starting from an initial position of 300 µm. Due to the need for conservative adjustment of control parameters from simulation to experiment, on the other hand, excessively high control gains can excite unmodeled dynamic components and cause chattering phenomena. Therefore, the control gain in the experiment is smaller than that in the simulation study. The final tuned parameters used for the Adaptive Fuzzy Fixed-Time Control (AF-FxT-C) are listed in the corresponding column of Table 2.

**Figure 5:**
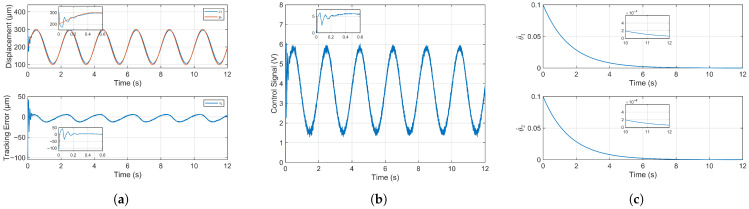
Experimental results. (**a**) Trajectory tracking control result and tracking error of experimental study. (**b**) Control signal in experimental study. (**c**) Estimation value of adaptive law in experimental study.

Figure 5a illustrates the tracking performance of the system output displacement (state $x_{1}$) against the reference signal, using feedback from a laser displacement sensor for closed-loop control. The system demonstrates rapid convergence and stabilization near the reference trajectory. The experimental results yield a root mean square error (RMSE) of 11.3675 µm in the first cycle and 7.0044 µm thereafter. The stabilized RMSE constitutes 3.5% of the total motion range, indicating high-precision tracking. The system’s rapid convergence is consistent with simulation predictions, though the experimental errors are slightly higher, which can be attributed to unmodeled real-world dynamics such as external disturbances or sensor noise.

The temporal variation of the control voltage is shown in Figure 5b. The voltage exhibits rapid changes during the initial transient phase, followed by stable sinusoidal fluctuations, which confirms the rapid stabilization of the system. In contrast to the simulation results, the experimental control voltage displays slight chattering and ultimately manifests a waveform containing minor glitches. These imperfections are likely attributable to unmodeled hardware dynamics, such as amplifier noise or discretization effects in the digital controller.

The evolution of the adaptive law estimates is presented in Figure 5c. Their rapid convergence demonstrates the stability of the adaptation mechanism and, by extension, the closed-loop system. Following convergence, the parameter estimates stabilize at magnitudes on the order of $10^{-4}$. Furthermore, the estimates remain bounded throughout the entire process; this is consistent with theory and simulation, which is a key requirement for system stability.

A comparison between simulation and experimental results revealed a consistent offset in the steady-state error. Furthermore, the third experiment demonstrated that different initial voltage settings induced a creep phenomenon in the piezoelectric injector during the initial control stage. This creep behavior resulted in slight variations in the steady-state error offset across different initial conditions. Therefore, it is confirmed that the observed steady-state error offset in the experiments is attributable to the inherent creep phenomenon of the piezoelectric actuator.

### 6.2. Comparison with Three Other Control Methods

This section presents a comparative experimental analysis of the proposed adaptive fuzzy fixed-time control (AF-FxT-C) strategy against three other methods: adaptive fuzzy finite-time control (AF-FnT-C), fixed-time sliding mode control (FxT-SMC), and proportional-integral-derivative control (PID-C). The initial state is denoted as “IS”. The comparison is conducted to evaluate performance under consistent conditions, though layout constraints necessitate the use of these acronyms in the subsequent figures and discussion.

**Figure 6:**
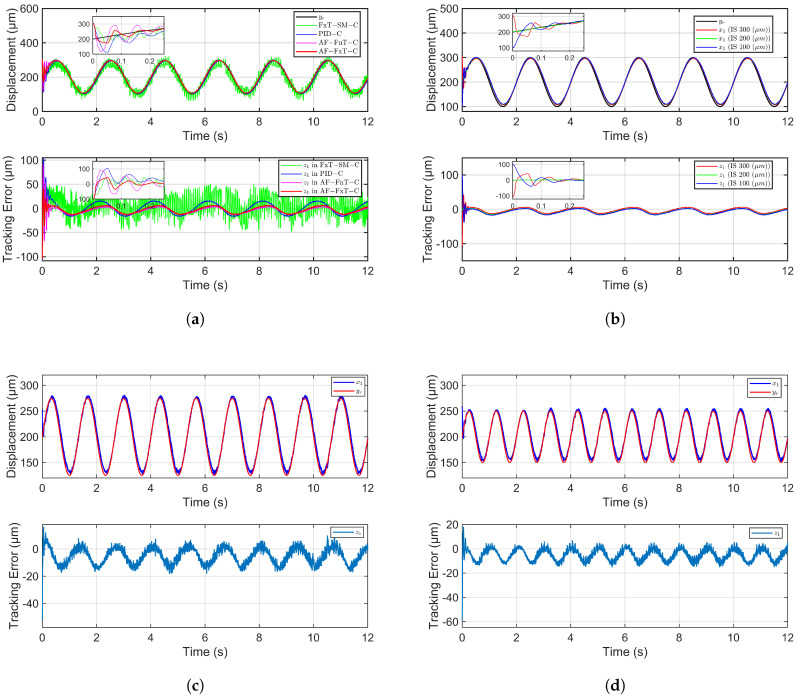
Experimental results of different control schemes. (**a**) Trajectory tracking control results of four control methods. (**b**) Adaptive fuzzy fixed-time trajectory tracking control results under different initial states. (**c**) Adaptive fuzzy fixed-time trajectory tracking control results under lower frequency and larger amplitude. (**d**) Adaptive fuzzy fixed-time trajectory tracking control results under higher frequency and smaller amplitude.

The control parameters for the AF-FnT-C and AF-FxT-C strategies required adjustment, as identical gains induced oscillations. Their carefully tuned parameters are detailed in Table 2. With the initial state set uniformly to 300 µm, Figure 6a presents the trajectory tracking performance and corresponding tracking errors for all four control methods. The root mean square error (RMSE) of the first cycle is used to characterize the initial tracking error and reflects the settling time, while the RMSE of the remaining duration characterizes the steady-state error. Due to the consistent adjustment of the initial position bias and reference signal, a smaller RMSE in the first cycle can represent a smaller settling time of the system. A quantitative comparison of these RMSE metrics for the different controllers is provided in Table 3.

It is noteworthy that the tracking error of the proposed AF-FxT-C during the first cycle is significantly smaller than that of all other methods. Quantitatively, AF-FxT-C reduced the initial RMSE by 35.4% compared to AF-FnT-C, by 53.1% compared to FxT-SMC, and by 45.3% compared to PID-C. This superior performance indicates that AF-FxT-C achieves the fastest convergence speed when the initial state exhibits a 100-micrometer deviation. Furthermore, AF-FxT-C also maintains the smallest steady-state error. During the stabilized phase, it reduced the RMSE by 8.3% versus AF-FnT-C, by 71.6% versus FxT-SMC, and by 37.0% versus PID-C.

These experimental results align well with theoretical expectations. When the initial state deviates significantly from the reference signal, the high-order error terms in the fixed-time controller exert a stronger effect, enabling AF-FxT-C to achieve faster convergence than the finite-time AF-FnT-C. Conversely, the industrially prevalent PID algorithm struggles to balance the competing demands of rapid stabilization and oscillation suppression under large initial deviations. Although FxT-SMC also possesses the fixed-time convergence property, it is inherently plagued by the chattering phenomenon characteristic of sliding mode control. Chattering occurs when the state crosses the sliding surface. To reduce chattering without modifying the algorithm, the switching gain should be decreased. Otherwise, more sophisticated algorithms should be designed to avoid chattering, such as replacing the sign function with a saturation function. In fact, experimental factors like measuring noise, data acquisition, and control signal amplification all exacerbate the chattering phenomenon. This chattering results in excessive steady-state errors and poses a potential risk of damage to the piezoelectric actuator.

The proposed AF-FxT-C method successfully solves the critical challenge of achieving rapid system stabilization from arbitrary initial conditions. This capability is particularly vital for piezoelectric microinjection systems, which are characterized by inherent hysteresis nonlinearity and necessitate frequent start-stop operating cycles.

### 6.3. Comparison of AF-FxT-C in Different Initial States

The experiment was configured with three distinct initial positions: 300 µm, 200 µm, and 100 µm, tracking the same reference signal used in the first experiment. As shown in Figure 6b, we observe that the system achieves rapid stabilization across all initial states. The moment when the oscillation ends is taken as the settling time, and under the initial condition of IS 200 µm, the system is stable from the beginning. At the initial states of IS 300 µm and IS 100 µm, the settling time of the system is 0.29 s and 0.27 s, respectively. validating the theoretical principle that the upper bound of the settling time in fixed-time control is independent of initial conditions.

### 6.4. Trajectory Tracking at Higher Frequency and Smaller Amplitude

To further verify the robustness and adaptability of the proposed control method, two additional experiments were conducted with varying reference signal frequencies and amplitudes. The AF-FxT-C strategy was applied for trajectory tracking in both cases, with a fixed reference signal bias of 200 µm and an initial system position of 250 µm.

As shown in Figure 6c, for a reference signal of 0.75 Hz and 75 µm amplitude, the system achieved an RMSE of 9.0385 µm in the first cycle and 7.7289 µm thereafter. Subsequently, as depicted in Figure 6d, for a more demanding 1 Hz, 50 µm amplitude reference, the system maintained strong performance with an RMSE of 8.5737 µm in the first cycle and 6.9087 µm during the remaining time.

These results demonstrate that the system displacement can accurately track the reference signal under varying operational conditions. Consequently, the proposed AF-FxT-C method enhances the versatility of the piezoelectric microinjector, enabling its application in more demanding scenarios, such as rapid injections into smaller biological cells.

It is notable that to better track the high-frequency reference signal, we have increased the control gain, but this will cause chattering after the system stabilizes, as shown in Figure 6c,d. Excessive reference signal frequency can reduce control effectiveness and even lead to system instability.

## 7. Conclusions

This paper proposed an adaptive fuzzy fixed-time control (AF-FxT-C) strategy for piezoelectric microinjectors. An adaptive fuzzy system approximates the combined disturbances, including hysteresis, while a fixed-time controller, designed via backstepping, ensures convergence within a bounded time independent of initial conditions. Stability is guaranteed through Lyapunov analysis. Simulations and experiments validate the method’s performance. Using a 0.5 Hz, 200 µm reference signal with 100 µm initial offset, the controller achieved an RMSE of 11.3675 µm in the first cycle and 6.9937 µm at steady state. It significantly outperformed adaptive finite-time, sliding-mode, and PID controllers in both convergence speed and accuracy and showed consistent performance across different frequencies and amplitudes. The proposed approach enables precise, rapid control of piezoelectric microinjectors, supporting applications requiring high-speed and high-precision motion. Future work will explore integral action to suppress creep effects and further extend the controller’s applicability.

## Figures and Tables

**Table 1:** System settings and control parameters.

Parameter and Setting	Value
Reference input signal $y_{r}$	200+100sin(0.5∗3.14t) µm
$x_{1}\left(0\right)$	300 µm
$x_{2}\left(0\right)$	0 µm
${\hat{\theta }}_{1}\left(0\right)={\hat{\theta }}_{2}\left(0\right)$	0.1
$K_{11}=K_{12}=K_{21}=K_{22}$	3.4
$p_{1}=p_{2}=q_{1}=q_{2}$	2.5
$\iota_{1}=\iota_{2}$	0.8
$O_{1}=O_{2}$	0.082

**Table 2:** Control parameters of the four control methods.

Parameter	AF-FxT-C	AF-FnT-C	FxT-SM-C	PID-C
$K_{11}=K_{21}$	3.4	2.8	-	-
$K_{12}=K_{22}$	3.4	2.8	-	-
$p_{1}=q_{1}$	2.5	2.5	-	-
$p_{2}=q_{2}$	2.5	2.5	-	-
$\iota_{1}=\iota_{2}$	0.8	0.8	-	-
$O_{1}=O_{2}$	0.082	0.082	-	-
Switch gain $k_{1}$	-	-	0.018	-
Switch gain $k_{2}$	-	-	0.01	-
Sliding surface gain *c*	-	-	5	-
$K_{p}$	-	-	-	31,000
$K_{i}$	-	-	-	1,600,000
$K_{d}$	-	-	-	10

**Table 3:** RMSE of the four control methods.

Method	RMSE in First Cycle (µm)	RMSE in Remaining Time (µm)
AF-FxT-C	11.3675	7.0044
AF-FnT-C	17.6038	7.2180
FxT-SM-C	24.2286	24.5096
PID-C	20.7762	11.0993

## Data Availability

The data that support the findings of this study are available from the corresponding author upon reasonable request.
